# Assessment of Nutritional Practices of Mountain Runners before and during Competitions

**DOI:** 10.3390/nu16162588

**Published:** 2024-08-06

**Authors:** Jan Walczak, Wiktoria Staśkiewicz-Bartecka, Agnieszka Białek-Dratwa, Mateusz Grajek, Agata Kiciak, Agnieszka Bielaszka, Marek Kardas

**Affiliations:** 1 Department of Food Technology and Quality Evaluation, Department of Dietetics, Faculty of Public Health in Bytom, Medical University of Silesia in Katowice, Ul. Jordana 19, 41-808 Zabrze, Poland; jwalczak@sum.edu.pl (J.W.); akiciak@sum.edu.pl (A.K.); abielaszka@sum.edu.pl (A.B.); mkardas@sum.edu.pl (M.K.); 2 Department of Human Nutrition, Department of Dietetics, Faculty of Public Health in Bytom, Medical University of Silesia in Katowice, Ul. Jordana 19, 41-808 Zabrze, Poland; abialek@sum.edu.pl; 3 Department of Public Health, Department of Public Health Policy, Faculty of Public Health in Bytom, Medical University of Silesia in Katowice, Ul. Piekarska 18, 41-902 Bytom, Poland; mgrajek@sum.edu.pl

**Keywords:** nutritional habits, dietary practices, sports nutrition, endurance athletes, performance nutrition, race nutrition

## Abstract

Mountain running, distinct from traditional road running, involves natural trails with significant elevation changes. This study aims to analyze dietary and supplementation practices among advanced and elite Polish mountain runners. Conducted from May to October 2023, this study included 36 participants (13 women, 23 men) aged 21–43 years. A custom questionnaire assessed nutrition two days before and during a competition, focusing on macronutrient intake, hydration, and supplementation. Statistical analyses were performed to compare advanced and elite athletes. Participants consumed an average of 3164.14 kcal two days before and 3176.97 kcal the day before the competition. Carbohydrate intake averaged 7.69 g/kg two days before and 7.64 g/kg the day before the race, potentially insufficient for optimal glycogen stores. Protein intake was adequate, averaging 1.63 g/kg two days before and 1.73 g/kg the day before the race. Fluid intake averaged 2811.25 mL two days before and 2891.80 mL the day before the race. During races, carbohydrate intake averaged 58.56 g/h, with variations based on race duration. Most participants used isotonic drinks and water for hydration. Mountain runners generally adhere to proper nutrition guidelines, with adequate protein and fluid intake. However, their carbohydrate intake may be insufficient for longer races. Higher carbohydrate intake during exercise could provide additional benefits.

## 1. Introduction

Mountain running is an increasingly popular form of physical activity. Unlike traditional road running, it occurs on natural trails and terrains, including mountains or hills [1]. Courses typically consist of forest trails, paths, and dirt roads, with minimal use of paved roads, which should not exceed 20–25% of the total race distance. A key feature is the significant elevation gain, with an average elevation change of at least 5% relative to the total course length [2,3]. Events are organized with increasing frequency, and the number of participants continues to grow [1].

Depending on the course’s nature and length, designated feeding stations are set up for assistance, such as exchanging water bottles or providing food. Organizers typically offer food and drinks at these stations, including fresh fruits, dried fruits, cookies, warm soups, and isotonic drinks or water [2,3]. Assistance outside designated points is usually prohibited, and essential safety gear may be mandated or recommended, such as mobile phones, thermal blankets, and rain jackets or pants [3,4].

Mountain running covers various distances, from short, several-kilometer races to vertical races (uphill), half-marathons, marathons, ultra, and multi-stage races [5]. It features more challenging terrain, significant elevation changes, irregular surfaces, and variable weather conditions compared to road running. Courses are often laid out on unpaved roads and mountain trails [3,6].

Athletes aim to progress and test their limits, with varying goals and definitions of success. Achieving results and improving sports performance involve optimizing factors like talent, genetic makeup, training programs, mental resources, motivation, body composition, nutrition, and hydration [7]. Proper nutritional preparation enables athletes to perform intense efforts, reduce the risk of injuries and illnesses, and improve recovery [8,9].

Energy requirements refer to the amount of energy needed to maintain body mass and composition, thermogenesis, metabolism of consumed nutrients, and physical activity. The basic metabolic rate (BMR) is the minimum energy required to sustain basic physiological functions at rest, influenced by age, sex, physiological state, body size, and composition, while the total energy expenditure (TEE) is calculated by multiplying the BMR by the physical activity level (PAL) [10,11].

Carbohydrates are the main energy source for the body and essential for energy metabolism. For athletes, the recommended intake is 5 to 12 g per kilogram of body mass per day, depending on the intensity of physical activity [12]. Strategies for carbohydrate intake before, during, and after exercise aim to optimize energy availability and improve performance. Recommendations include consuming high-carbohydrate meals and snacks before exercise, carbohydrate loading before competitions, and appropriate carbohydrate intake during and after exercise to maintain energy levels and support recovery [12,13].

Fats provide concentrated energy, essential fatty acids, and fat-soluble vitamins. They should cover 20–35% of the energy needs of athletes, with a balanced intake of saturated and unsaturated fats [12]. Protein is crucial for muscle repair, recovery, and overall health. Recommendations for protein intake vary based on activity level, with higher amounts needed for athletes. The quality and composition of protein, including essential amino acids, are important for optimal health and performance [14].

Proper hydration is vital for maintaining performance and health. Fluid needs vary based on diet, weather, and physical activity. Athletes should plan their hydration strategy, including pre-exercise hydration, fluid intake during exercise, and post-exercise rehydration. Monitoring hydration status through urine color, osmolality, and body weight changes helps manage fluid needs. Dehydration can impair performance, increase the risk of heat-related issues, and cause injuries [15].

Sports drinks containing carbohydrates and electrolytes help prevent dehydration and provide energy during exercise. They come in hypotonic, isotonic, and hypertonic forms, each suited for different exercise durations and intensities. Proper hydration strategies ensure continuous fluid intake and effective rehydration [16].

Supplements are used to improve performance, fill nutritional gaps, and support recovery. While they play a small role compared to factors like training and genetics, supplements can provide a competitive edge. They are categorized based on their evidence of effectiveness and safety, with recommendations varying by individual needs and goals [17,18,19].

The primary goal of this study is to analyze the dietary and supplementation practices of mountain runners participating in competitions. Specific objectives include evaluating the quality and quantity of nutrition in the two days leading up to the event, assessing pre-race nutrition strategies, analyzing in-race nutrition, and evaluating supplementation practices. Proper nutrition and hydration strategies are essential for endurance sports performance and athlete well-being, and this study aims to provide insights into these practices among mountain runners.

## 2. Materials and Methods

### 2.1. Research Design

This study was conducted from May to October 2023 and included mountain runners competing at advanced and elite levels. A dedicated sampling method was used to select the sample, ensuring that it represented the characteristics and specific experiences relevant to the research topic. The precise selection criteria, such as the type of sport and competition level, were crucial for achieving the study’s objectives.

The research was conducted during the 2023 competition season. Participants in this study were informed about the purpose of the study and its anonymity and were asked to accept the rules of data sharing. Information about informed and voluntary participation in this study was at the beginning of the questionnaire. The World Medical Association’s Declaration of Helsinki [20] guided the conduct of this study. This study was approved by the Bioethics Committee of the Silesian Medical University in Katowice (BNW/NWN/0043-3/641/35/23, date of approval: 22 September 2023) in light of the Law of 5 December 1996, on the Profession of Physician and Dentist (Journal of Laws 2016, item 727).

### 2.2. Participants

Participants were informed about this study’s purpose and anonymity and were asked to agree to the data-sharing terms. Information about voluntary and informed participation was provided at the beginning of the questionnaire. The study involved 36 participants, including 13 women and 23 men aged 21 to 43 years, with an average age of 31.4 ± 6.6 years. All participants were Polish nationals. The participants were divided into two groups based on their sport level: Overall, 66.67% (n = 24) were at the elite level (EL), while 33.33% (n = 12) were at the advanced level (AL). The majority of the study participants, 91.67% (N = 33), had a normal body weight. Underweight (BMI ≤ 18.5) participants constituted 8.33% (N = 3) of the group. The characteristics of the study participants are shown in Table 1.

The inclusion criteria for this study were as follows: (1) consent to participate in the study; (2) being at least 18 years old; (3) participation in mountain running competitions during the 2023 competition season, focusing on active mountain runners; (4) no injuries that prevented training for at least 7 days in the two months preceding the study, ensuring participants were in good physical condition and able to maintain consistent training, minimizing variability in the data due to recent injuries, and reflecting the experiences of healthy, active runners; and (5) a minimum sport level defined as advanced, based on the Rate My Trail ranking of mountain runners, which provides more reliable data on the practices and needs of professional athletes, as opposed to beginners who may have different training and nutritional requirements [21].

The exclusion criteria were as follows: (1) incorrectly or (2) incompletely filled-out questionnaires, to ensure the accuracy, reliability, and integrity of the data collected.

### 2.3. Research Tools

The questionnaire consisted of three parts: The first part included information about this study and instructions with an example of correctly filling out the tables. The second part consisted of metric questions regarding cooperation with a nutritionist, using a specific nutritional model; following an elimination diet; adjusting nutrition to physical activity; using carbohydrate loading protocols, dietary supplements, and tables concerning the two days preceding the competition; and questions about the specifics of the running event, respondent metrics, effort difficulty scale, self-assessment, gastrointestinal problems, or other ailments during the effort. The third part contained current recording method tables for three consecutive days—two days preceding the competition and the competition day, excluding post-race nutrition.

The method used to obtain the results was CAWI (Computer-Assisted Web Interview); the survey was conducted using an online form. Google Forms platform was used for data collection due to its ease of use, availability, and ability to customize the questionnaire to the study’s needs. The questionnaire was made available in Google Forms and then sent to a group of mountain runners via social media and email, with the help of the Tatra Running Festival organizer. This way, data from races of varying lengths, elevations, and effort durations were collected.

To increase the reliability of the results obtained through the current recording method, participants were asked to record daily data on consumed products both before and during the competition. This was to enhance the accuracy of the collected information. For recording food and fluid intake, participants were instructed to use precise weights.

Energy and macronutrient intake analysis was conducted using the Kcalmar Pro diet program, which contains a database from the “Food and Nutrition Tables”—Institute of Food and Nutrition, Warsaw 2017, 4th Edition [22]. Standardized recipes were created for products not included in the database, such as sports foods, according to the information on the food packaging.

The subjective effort severity was assessed using the modified Borg scale [23].

The Body Mass Index (BMI) was calculated by dividing the body mass (kg) by the square of the height (m). The results were used as the basis for comparing height-to-weight ratios for the European population according to WHO recommendations and guidelines [24].

### 2.4. Statistical Analysis

Statistical analyses were performed using Statistica v.13.3 (Stat Soft Poland, Krakow, Poland). For quantitative data presentation, mean values and standard deviations (X ± SD) were calculated; for qualitative data, percentage notation was used.

The normal distribution was checked using the Shapiro–Wilk test. The significance of differences between advanced and elite athletes and between male and female athletes was assessed using Student’s *t*-test for two independent samples or the Mann–Whitney U test for non-parametric groups. The Chi^2^ test of independence or Fisher’s exact test was used to compare nominal variables.

A significance level of *p* < 0.05 was applied as the criterion for statistical significance.

## 3. Results

### 3.1. Characteristics of the Sporting Events

The average effort duration for respondents during competitions was 255.39 ± 224.84 min (approximately 4 h 15 min ± 3 h 44 min). The shortest run, a vertical race, lasted 46 min, while the longest, an ultra-race, lasted 1140 min (19 h). Differences in effort duration between elite and advanced athletes were not statistically significant (*p* = 0.94).

Participants covered an average distance of 37.32 ± 31.40 km, with the shortest race being 8.00 km and the longest 162.00 km. The differences in course length between elite and advanced athletes were not statistically significant (*p* = 0.83). The average elevation gain on race courses was 2135.00 ± 1407.95 m. Differences in total ascent between advanced and elite groups were not statistically significant (*p* = 0.15).

Using the modified Borg scale, most respondents rated their fatigue as very high (8.77%, n = 5), while only one rated it as moderate (1.76%, n = 1). Detailed fatigue ratings are provided in Table 2.

The majority of respondents did not report gastrointestinal problems during the competition (80.56%; n = 29). The reported issues included cramps (5.5%; n = 2), stomach pain (5.5%; n = 2), discomfort (5.5%; n = 2), and nausea (2.78%; n = 1). More than half of the participants experienced a drop in energy during the competition (52.78%; n = 19), while thirteen individuals (36.11%) reported no issues.

### 3.2. Nutritional and Supplementation Behaviors of Study Participants

Nearly half of the respondents (44.44%; n = 16) had never used the services of a dietitian, while 27.78% (n = 10) had used such services in the past, and 19.44% (n = 7) were currently under dietary supervision. The majority (83.33%; n = 30) did not adhere to a specific nutritional model, with 13.89% (n = 5) following a vegetarian diet and 2.78% (n = 1) adhering to a pescatarian diet. Most participants (94.44%; n = 34) did not follow an elimination diet, with only 5.56% (n = 2) limiting their intake of lactose and gluten.

**Figure 1:**
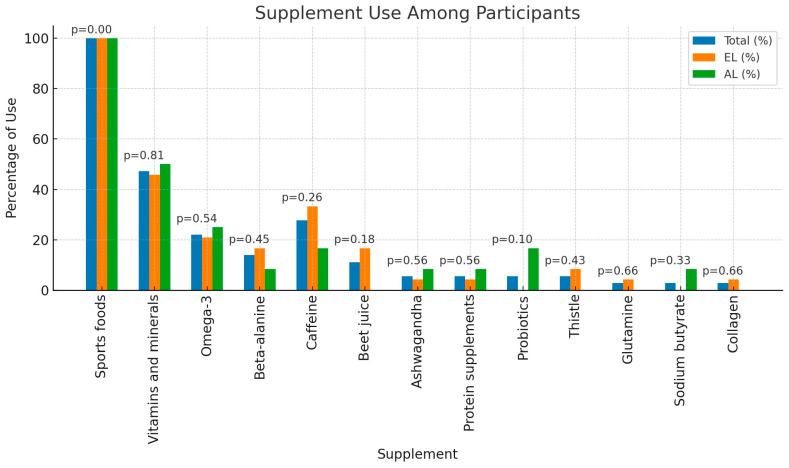
Supplements used by athletes by sport level; El—elite level; AL—advanced level (n = 36).

Nearly all respondents (94.44%; n = 34) adjusted their diet according to their physical activity, with only 5.56% (n = 2) not making such adjustments. Most respondents (55.56%; n = 20) did not use the carbohydrate loading protocol, while 44.44% (n = 16) reported using this strategy. Differences between elite and advanced athletes in the use of the carbohydrate loading protocol were not statistically significant (*p* = 0.81). All participants used dietary supplements, with detailed information on supplementation presented in Figure 1.

### 3.3. Nutrition in the Days Leading up to the Competition

Two days before the competition, respondents consumed an average of 3164.14 ± 651.59 kilocalories, and 3176.97 ± 939.66 kilocalories on the day before. The difference in caloric intake between women and men was statistically significant both the day before (*p* < 0.001) and two days before the race (*p* < 0.001). Detailed information on the intake of energy, protein, fats, and carbohydrates per kilogram of body mass per day, considering the level of athletic performance, is presented in Table 3.

The quantitative intake of carbohydrates per kilogram of body mass did not differ statistically between individuals who used the carbohydrate loading protocol and those who did not, both two days before the competition (*p* = 0.75) and the day before the start (*p* = 0.41). The average fiber intake among the respondents was 33.17 ± 16.15 g two days before the start and 28.41 ± 11.85 g on the day before with no significant difference between the advanced and elite groups on either day (*p* = 0.60; *p* = 0.99).

Respondents consumed an average of 2811.25 ± 642.84 mL of fluids two days before the start, and 2891.80 ± 806.96 mL the day before the competition. Fluid intake differences between the advanced and elite groups were not statistically significant (*p* = 0.09; *p* = 0.81).

Most respondents performed aerobic running training before the competition, with 72.22% (n = 26) training two days before and 61.11% (n = 22) the day before the start. It was shown that 30.77% of respondents performed a workout of more than 60 min two days before the start and as many as 45.45% the day before the start. No significant differences were found between the advanced and elite groups either two days before (*p* = 0.44) or the day before the race (*p* = 0.09).

### 3.4. Nutrition before the Race

Almost all respondents (97.22%; n = 35) consumed a pre-race meal, with no statistically significant difference between the elite and advanced groups (*p* = 0.66). Among those who had a pre-race meal, 65.71% (n = 23) consumed it between two and four hours before the effort. Detailed information regarding the pre-race meal is provided in Table 4.

**Figure 2:**
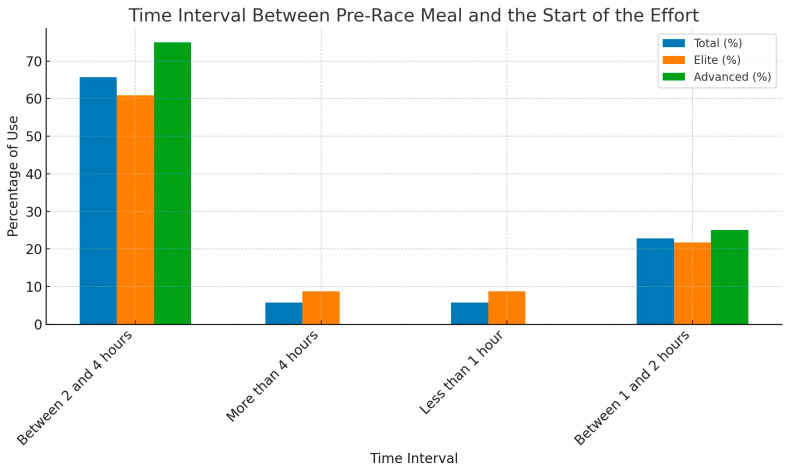
The time interval between the pre-race meal and the start of the effort, categorized by total, elite level (EL) and advanced level (AL) (n = 23).

Among those who consumed a pre-race meal, the majority (65.71%, n = 23) ate it between two and four hours before the effort. Detailed information is shown in Figure 2.

**Figure 3:**
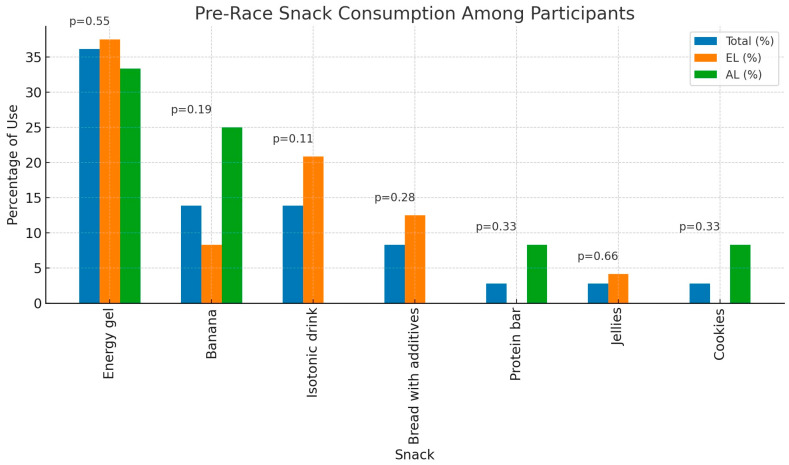
Most frequently consumed pre-race snacks; El—elite level; AL—advanced level (n = 26).

Most respondents (72.22%; n = 26) consumed a pre-race snack, with no statistically significant difference in frequency between performance levels (*p* = 0.44). The average carbohydrate content in the pre-race snack was 32.37 ± 16.42 g, with no significant differences between the elite and advanced groups (*p* = 0.26). Detailed information regarding pre-race snack products is provided in Figure 3.

### 3.5. Nutrition and Hydration during Race

Nearly all respondents (88.98%; n = 32) utilized nutritional support during exercise. Participants in events lasting less than one hour did not use energy supplementation (100.00%; n = 3). Among those whose effort lasted more than one hour but less than two hours, only one did not use energy supplementation during the event (14.29%; n = 1). All respondents in races lasting more than two hours supplemented their energy intake during exercise (100.00%; n = 26). Most respondents using energy supplementation consumed energy gels (96.98%; n = 31), while 25.00% (n = 8) consumed fruits such as watermelon, bananas, and oranges. Differences in the consumption of specific products between the elite and advanced groups were not statistically significant (*p* < 0.05). Detailed information regarding carbohydrate intake during the race is presented in Table 5.

Almost all respondents (94.44%; n = 34) consumed fluids during exercise, with an average intake of 488.11 ± 252.69 mL per hour. The difference in fluid consumption between the elite and advanced groups was not statistically significant (*p* = 0.94). The highest average fluid intake per hour was reported by runners whose effort lasted more than three hours (525.65 ± 261.01 mL per hour). Detailed information on the amount of fluid consumed is presented in Table 6.

**Figure 4:**
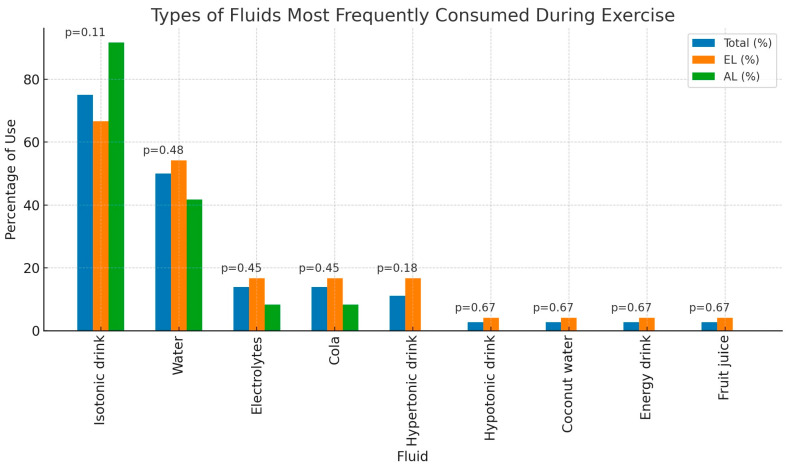
Types of fluids most frequently consumed during exercise; El—elite level; AL—advanced level (n = 36).

The most frequently chosen type of fluid among participants using hydration strategies during the competition was isotonic drinks (75.00%; n = 27), with half of the respondents also consuming water (50.00%; n = 18). The differences between the advanced and elite groups regarding the frequency of selecting a particular type of fluid were not statistically significant. Detailed information on the types of fluids consumed during the competition is provided in Figure 4.

## 4. Discussion

This study assessed the nutrition of advanced and elite-level mountain runners from Poland over the two days preceding the competition and on race day, using the current recording method. This study included 26 men and 13 women, with an average age of 31.39 ± 6.65 years. Almost all respondents (91.67%) in the current study had a normal body mass (WHO classification), with 8.33% of respondents being underweight [24,25].

Melo et al. [26] studied 48 Colombian mountain runners, with men averaging a height of 168.1 ± 6.2 cm, a body mass of 64.87 ± 7.53 kg, and a BMI of 22.99 ± 2.61 kg/m^2^. Women averaged a height of 158.9 ± 6.2 cm, a body mass of 53.37 ± 3.64 kg, and a BMI of 20.89 ± 1.53 kg/m^2^. The current study’s participants had a higher BMI, body mass, and height compared to the other groups.

Despite significant individual differences in calorie, protein, carbohydrate, fat, fiber, and fluid intake, the nutrition of the participants could be considered appropriate.

On both days before the competition, carbohydrate intake values were too low to optimize muscle glycogen stores compared to recommendations for endurance athletes participating in competitions lasting more than 90 min [13,20].

In the study by Carlsohn and Müller [27] on six elite mountain runners, it was observed that participants consumed too little carbohydrates in the days preceding the competition to meet the requirements of the carbohydrate loading protocol and thus ensure optimal muscle glycogen concentration on the day of the competition [27]. However, the current study did not collect data from the third and fourth days before the competition, so it cannot be conclusively stated that participants who declared the use of the carbohydrate loading protocol did not follow the carbohydrate intake recommendations to optimize muscle glycogen concentration.

The recommended protein intake for athletes is 1.2–2.0 g/kg body weight per day. Such values are recommended by expert societies, including AND (Academy of Nutrition and Dietetics), DC (Dietitians of Canada), and ACSM (American College of Sports Medicine) [20]. In the current study, the recommended protein intake was met [11].

Almost all respondents (97.22%, n = 35) consumed a pre-race meal, with the majority (88.57%, n = 31) eating it within 1–4 h before the race. The average carbohydrate content of these meals was 1.38 ± 0.54 g/kg body weight. It is recommended that a pre-race meal be consumed within 1 to 4 h before starting exercise, with a carbohydrate content of 1–4 g/kg body weight. Additionally, the fat, protein, and fiber content should be reduced to avoid potential gastrointestinal issues. Most study participants followed these recommendations [20]. In a study by Atkinson et al. involving 257 marathon runners, participants consumed an average of 1.7 ± 0.8 g/kg body weight of carbohydrates in their pre-race meal [28].

All participants in the current study used dietary supplements. The most commonly used supplements were sports foods, vitamin–mineral preparations, and caffeine. These supplements are categorized in Group A by the Australian Institute of Sport (AIS), indicating they have the most scientific evidence supporting their use in sports [16]. Jiménez-Alfageme et al. [29] conducted a study involving 357 Spanish mountain runners; almost all participants (93.84%) used dietary supplements, with the most commonly used being sports foods and caffeine. It was observed that athletes at a higher level of advancement used more dietary supplements [29].

In another analysis by Jiménez-Alfageme et al. [30] involving 72 mountain runners, it was observed that men used dietary supplements more often than women, and overall, dietary supplements were used by 87.5% of respondents. The most commonly used supplements were sports food, caffeine, and magnesium. Compared to the above studies, respondents in the current study used dietary supplements more frequently, but the differences in usage were not statistically significant based on the level of advancement. In both the cited analyses and the current study, the most commonly used supplements were sports food, caffeine, and vitamin–mineral preparations [30]. Dietary supplements are practically an inseparable element from mountain running and were used by all study participants. Therefore, caution should be exercised when using them due to the possibility of contamination with doping substances [20].

Carbohydrate intake values during exercise are within the recommended ranges for endurance athletes [20,31]. Other studies [14,27,32] have observed similar or lower carbohydrate intakes, indicating the need for potentially higher values to reduce muscle damage and improve performance.

Similar values of fluid intake as in our study were reported by Martinez et al. [32] and Carlsohn and Müller [13].

In the analysis by Martinez et al. [32], the average fluid intake among respondents was 425.0 ± 249.6 mL/h. Due to the lack of data on individual sweating rates and changes in body weight, the authors considered the average fluid intake appropriate [32]. In the study by Carlsohn and Müller [27], the average fluid intake was 449 ± 376 mL/h. In this case, the researchers were unable to determine whether these amounts were sufficient to prevent dehydration exceeding a 2% body weight loss due to the lack of data on body weight changes and individual sweating rates [27].

In the current study, no data on individual sweating rates and body weight changes among athletes were collected either. Additionally, participants took part in various sports events, where weather conditions could differ significantly. Therefore, it cannot be stated whether the average fluid intake was sufficient to prevent dehydration exceeding a 2% body weight loss.

The current study has some limitations. Firstly, participants took part in various running events, which varied in terms of course specifics, altitude, and weather conditions. Secondly, the main food data collection tool was the current record-keeping method, which may have some limitations due to memory gaps of the respondents and inaccurate estimation of product portions. Additionally, the current record-keeping method was problematic for respondents in the days preceding the competition and on the day of the race (due to lack of time and other race-related responsibilities), which contributed to difficulties in recruiting respondents and rejecting some incorrectly filled questionnaires.

This study was conducted using a carefully selected sample that represented the specific characteristics and experiences of advanced and elite-level mountain runners. Furthermore, participants were asked to record daily data on consumed products before and during the competition, which enhanced the accuracy of the obtained information. It is worth emphasizing that, according to the review of the current literature, this is the first study evaluating the nutritional behaviors encompassing preparation and participation in competitions among elite and professional mountain runners.

## 5. Conclusions

Data analysis showed that mountain runners participating in sports competitions of varying durations generally have proper nutrition, such as consuming an appropriate amount of protein, kilocalories, and fluids in the days leading up to the start. Differences in nutrition between the elite and advanced groups were not statistically significant. The carbohydrate intake before the competition was likely too low to optimize muscle glycogen levels among those whose competitions exceeded 90 min. The average amount of carbohydrates consumed by respondents during exercise was higher than in available studies involving mountain and long-distance runners. For participants whose effort lasted less than 180 min, carbohydrate values per hour corresponded with current recommendations for endurance athletes. However, for those whose competitions lasted longer than 180 min, carbohydrate intake was probably too low. It is worth noting that in light of the latest scientific reports, higher amounts of carbohydrates consumed during exercise can bring additional benefits. Advanced and elite-level mountain runners demonstrate high nutritional awareness and use nutrition strategies consistent with recommendations for endurance athletes, which may contribute to their better sports performance. However, further research is needed to better understand the individual differences and nutritional needs of this specific group of athletes.

## Figures and Tables

**Table 1:** Characteristics of study participants, with a comparison between EL and AL (n = 36).

Variable	Level	Gender	Average	Median	Standard Deviation	*p*-Value
Age [years]	Total (n = 36)	Total (n = 36)	31.39	31.00	6.65	0.66
		W (n = 13)	32.20	30.00	5.53	
		M (n = 23)	30.90	30.00	7.28	
	EL (n = 24)	Total (n = 24)	31.05	31.00	6.49	
		W (n = 10)	32.50	33.5	6.06	
		M (n = 14)	30.00	29.5	6.80	
	AL (n = 12)	Total (n = 12)	32.08	31.5	7.19	
		W (n = 3)	31.30	32	4.04	
		M (n = 9)	32.30	31	8.17	
Body mass [kg]	Total (n = 36)	Total (n = 36)	64.22	65.00	9.64	0.13
		W (n = 13)	54.49	53.00	4.74	
		M (n = 23)	69.72	71.00	6.94	
	EL (n = 24)	Total (n = 24)	62.50	63.25	8.43	
		W (n = 10)	55.34	54.00	5.10	
		M (n = 14)	67.61	67.50	6.34	
	AL (n = 12)	Total (n = 12)	67.67	71.00	11.31	
		W (n = 3)	51.67	52.00	1.53	
		M (n = 9)	73.00	74.00	6.87	
Body height [m]	Total (n = 36)	Total (n = 36)	1.74	1.73	0.09	0.56
		W (n = 13)	1.66	1.65	0.05	
		M (n = 23)	1.79	1.81	0.07	
	EL (n = 24)	Total (n = 24)	1.74	1.71	0.10	
		W (n = 10)	1.66	1.64	0.06	
		M (n = 14)	1.79	1.81	0.08	
	AL (n = 12)	Total (n = 12)	1.76	1.77	0.08	
		W (n = 3)	1.65	1.65	0.00	
		M (n = 9)	1.79	1.81	0.06	
BMI [kg/m^2^]	Total (n = 36)	Total (n = 36)	21.01	20.99	1.67	0.052
		W (n = 13)	19.84	19.57	1.24	
		M (n = 23)	21.68	21.91	1.53	
	EL (n = 24)	Total (n = 24)	20.63	20.83	1.32	
		W (n = 10)	20.09	20.66	1.29	
		M (n = 14)	21.02	21.10	1.24	
	AL (n = 12)	Total (n = 12)	21.77	22.25	2.08	
		W (n = 3)	18.98	19.10	0.56	
		M (n = 9)	22.70	22.86	1.40	

W—women; M—men; El—elite level; AL—advanced level.

**Table 2:** Respondents’ fatigue ratings using the modified Borg scale.

Borg Scale Rating	Definition of Rating	n (%)
3	Moderate fatigue, moderate breathlessness	1 (1.76%)
4	Quite high fatigue, relatively heavy breathlessness	1 (1.76%)
5	High fatigue, heavy breathlessness	3 (5.26%)
6	High fatigue, heavy breathlessness	5 (8.77%)
7	Very high fatigue, very heavy breathlessness	5 (8.77%)
8	Very high fatigue, very heavy breathlessness	5 (8.77%)
9	Very high fatigue, very heavy breathlessness	8 (14.04%)
10	Very, very high fatigue, almost maximal breathlessness	6 (10.53%)
10+	Maximal fatigue, unbearable breathlessness	2 (3.51%)

**Table 3:** Macronutrient content on the days leading up to the race by group according to performance level (n = 36).

Variable		Level	Average	Median	Standard Deviation	*p*-Value
Energy [kcal/kg b.w.]	Two days before the race	Total (n = 36)	49.80	48.00	10.60	0.119
		EL (n = 24)	45.90	47.10	5.13	
		AL (n = 12)	51.80	49.9	12.00	
	The day before the race	Total (n = 36)	50.40	48.10	16.7	0.123
		EL (n = 24)	53.40	51.70	18.50	
		AL (n = 12)	44.30	45.10	10.3	
Protein [g/kg b.w.]	Two days before the race	Total (n = 36)	1.63	1.53	0.50	0.62
		EL (n = 24)	1.78	1.64	0.50	
		AL (n = 12)	1.65	1.67	0.36	
	The day before the race	Total (n = 36)	1.73	1.70	0.58	0.45
		EL (n = 24)	1.78	1.73	0.65	
		AL (n = 12)	1.63	1.62	0.42	
Fats [g/kg b.w.]	Two days before the race	Total (n = 36)	1.43	1.28	0.56	0.55
		EL (n = 24)	1.47	1.23	0.63	
		AL (n = 12)	1.35	1.29	0.39	
	The day before the race	Total (n = 36)	1.51	1.39	0.92	0.33
		EL (n = 24)	1.62	1.45	1.04	
		AL (n = 12)	1.27	1.23	0.59	
Carbohydrates [g/kg b.w.]	Two days before the race	Total (n = 36)	7.69	7.35	2.05	0.24
		EL (n = 24)	8.04	7.64	2.30	
		AL (n = 12)	7.00	7.07	1.23	
	The day before the race	Total (n = 36)	7.64	7.62	2.35	0.09
		EL (n = 24)	8.10	7.89	2.57	
		AL (n = 12)	6.71	7.03	1.54	

El—elite level; AL—advanced level.

**Table 4:** Macronutrient content in the pre-race meal (n = 35).

Variable	Level	Average	Median	Standard Deviation	*p*-Value
Carbohydrates [g/kg b.w.]	Total (n = 35)	1.38	1.36	0.54	0.18
	EL (n = 23)	1.47	1.47	0.61	
	AL (n = 12)	1.20	1.23	0.33	
Protein [g]	Total (n = 35)	12.48	10.00	6.95	0.49
	EL (n = 23)	11.90	11.00	6.00	
	AL (n = 12)	13.61	10.00	8.69	
Fats [g]	Total (n = 35)	10.60	9.00	9.45	0.66
	EL (n = 23)	9.49	8.60	8.37	
	AL (n = 12)	12.73	9.20	11.33	

El—elite level; AL—advanced level.

**Table 5:** Average carbohydrate intake in grams: overall and per hour of exercise based on the duration of the race (n = 32).

	Level	Average	Median	Standard Deviation	*p*-Value
Average Carbohydrate Intake During the Race [g/h]	Total (n = 32)	58.56	57.14	28.61	0.89
	EL (n = 22)	59.04	53.74	30.39	
	AL (n = 10)	57.65	57.16	26.04	
Between 1 and 2 h [g/h]	Total (n = 6)	35.21	24.42	27.06	00.92
	EL (n = 5)	35.95	24.42	28.92	
	AL (n = 1)	33.37	33.37	32.21	
Between 2 and 3 h [g/h]	Total (n = 5)	52.80	48.42	18.21	-
	EL (n = 4)	44.91	44.52	5.22	
	AL (n = 1)	84.35	84.35	-	
More than 3 h [g/h]	Total (n = 21)	67.74	67.00	27.13	0.34
	EL (n = 13)	72.26	69.48	29.19	
	AL (n = 8)	60.38	61.90	23.28	

El—elite level; AL—advanced level.

**Table 6:** Fluid intake during exercise in milliliters per hour based on the duration of exercise (n = 34).

	Average	Median	Standard Deviation
Less than one hour [mL/h] (n = 1)	63.83	63.83	-
Between 1 and 2 h [mL/h] (n = 7)	470.60	495.41	289.02
Between 2 and 3 h [mL/h] (n = 5)	439.82	436.37	70.87
More than 3 h [mL/h] (n = 21)	525.65	491.80	261.01

## Data Availability

The data presented in this study are available upon request from the corresponding author. The data are not publicly available due to privacy.
